# INFLAMMATION AND DOMAIN-SPECIFIC COGNITIVE PERFORMANCE: RESULTS FROM THE EINSTEIN AGING STUDY

**DOI:** 10.1093/geroni/igae098.3047

**Published:** 2024-12-31

**Authors:** Molly Wright, Erin Harrington, Jennifer Graham-Engeland, Martin Sliwinski, Richard Lipton, Mindy Katz, Christopher Engeland

**Affiliations:** The Pennsylvania State University, University Park, Pennsylvania, United States; University of Wyoming, Laramie, Wyoming, United States; Pennsylvania State University, University Park, Pennsylvania, United States; Pennsylvania State University, University Park, Pennsylvania, United States; Albert Einstein College of Medicine, Bronx, New York, United States; Albert Einstein College of Medicine, Bronx, New York, United States; The Pennsylvania State University, University Park, Pennsylvania, United States

## Abstract

Inflammation may serve as a biological precursor to the development of mild cognitive impairment (MCI) and dementia. Although interleukin (IL)-6 may be more strongly associated with executive functioning (EF) or processing speed (PS) measures versus other cognitive domains, this has not been assessed simultaneously in a structural equation modeling (SEM) framework among older adults. The present study sought to address this gap. As part of the ongoing Einstein Aging Study, older adults (n=159, 65.4% female, 49.7% non-Hispanic White) without MCI at baseline completed neurocognitive testing and had two blood draws spaced two weeks apart at their baseline wave of data collection. Inflammation was assessed by averaging IL-6 levels obtained from these blood draws. Based on prior factor-analytic studies, a confirmatory factor analysis (CFA) was conducted that included an attention factor (Number Span Forward and Back, Verbal Fluency Test), memory factor (Free Selective Reminding Test, Logical Memory Test, Verbal Fluency Test), and EF/PS factor (Trail Making Test Parts A and B, Digit Symbol Test). A SEM model with pathways from IL-6 predicting cognitive factors was then specified, adjusting for relevant covariates based on fit statistics (age, years of education, sex, race/ethnicity). Both the CFA and SEM models fit the data well (RMSEA< 0.05, CFI>0.95). As expected, IL-6 was negatively associated with the EF/PS factor (β=-0.251, S.E.=0.073, p< 0.01). IL-6 was not associated with either the attention or memory factors (p>0.5). Based on these results, we predict that higher levels of IL-6 may predict nonamnestic MCI but not amnestic MCI.

